# The injury-induced myokine insulin-like 6 is protective in experimental autoimmune myositis

**DOI:** 10.1186/2044-5040-4-16

**Published:** 2014-08-04

**Authors:** Ling Zeng, Sonomi Maruyama, Kazuto Nakamura, Jennifer L Parker-Duffen, Ibrahim M Adham, Xuemei Zhong, Han-Kyu Lee, Henry Querfurth, Kenneth Walsh

**Affiliations:** 1 Whitaker Cardiovascular Institute, Boston University School of Medicine, 715 Albany Street, W611, Boston, MA 02118, USA; 2 Institute of Human Genetics, University of Göttingen, Göttingen, Germany; 3 Hematology Oncology Section, Boston University School of Medicine, 715 Albany Street, W611, Boston, MA 02118, USA; 4 Department of Neurology, Rhode Island Hospital, Brown University School of Medicine, 593 Eddy St, Providence, RI 02903, USA

**Keywords:** Insulin-like 6, Myositis, Autoimmune, T cells, Skeletal muscle, Myokine

## Abstract

**Background:**

The idiopathic inflammatory myopathies represent a group of autoimmune diseases that are characterized by lymphocyte infiltration of muscle and muscle weakness. Insulin-like 6 (Insl6) is a poorly characterized member of the insulin-like/relaxin family of secreted proteins, whose expression is upregulated upon acute muscle injury.

**Methods:**

In this study, we employed Insl6 gain or loss of function mice to investigate the role of Insl6 in a T cell-mediated model of experimental autoimmune myositis (EAM). EAM models in rodents have involved immunization with human myosin-binding protein C with complete Freund’s adjuvant (CFA) emulsions and pertussis toxin.

**Results:**

Insl6-deficiency in mice led to a worsened myositis phenotype including increased infiltration of CD4 and CD8 T cells and the elevated expression of inflammatory cytokines. Insl6-deficient mice show significant motor function impairment when tested with treadmill or Rotarod devices. Conversely, muscle-specific overexpression of Insl6 protected against the development of myositis as indicated by reduced lymphocyte infiltration in muscle, diminished inflammatory cytokine expression and improved motor function. The improvement in myositis by Insl6 could also be demonstrated by acute hydrodynamic delivery of a plasmid encoding murine Insl6. In cultured cells, Insl6 inhibits Jurkat cell proliferation and activation in response to phytohemagglutinin/phorbol 12-myristate 13-acetate stimulation. Insl6 transcript expression in muscle was reduced in a cohort of dermatomyositis and polymyositis patients.

**Conclusions:**

These data suggest that Insl6 may have utility for the treatment of myositis, a condition for which few treatment options exist.

## Background

Idiopathic inflammatory myositis is a category of autoimmune muscle disorders that include polymyositis, dermatomyositis and inclusion body myositis. Features common to all of the subtypes include muscle weakness, histological evidence of muscle inflammation and elevated levels of creatine kinase. However, each subset also has unique clinical, immunopathologic and histologic criteria [1]. Dermatomyositis is a vascular endothelial lesion associated with perimysial inflammation and perifascicular muscle fiber atrophy, whereas polymyositis is associated with injury to muscle fibers by aggressive immune cells which predominantly infiltrate the endomysium [2]. Macrophages, dendritic cells, and T cells are prominently present in muscle of the different myositis subgroups. In dermatomyositis, large numbers of helper T cells (CD4 T cells) are found within the perimysial, often perivascular areas. In polymyositis, activated cytotoxic T cells (CD8 T cells) surround and invade non-necrotic muscle fibers, while helper T cells are found at more distant parts of the infiltrates [3]. These inflammatory muscle diseases are rare and there are few effective treatment strategies for these patients [4].

Immune cells regulate skeletal muscle regeneration after myofiber damage [5,6]. Muscle damage stimulates massive infiltration of immune cells into the injury site, including neutrophils, macrophages, and T cells. These cells function in the phagocytosis of necrotic muscle fibers, express pro-inflammatory cytokines and participate in satellite cell activation [7]. We have previously demonstrated that Insl6 has muscle regenerative and anti-inflammatory activities in a cardiotoxin-induced model of severe muscle injury when expressed using an engineered adenovirus vector [8]. We therefore sought to extend these studies and explore the utility of Insl6 in the setting of inflammatory muscle injury using a rodent model of myositis.

In the current study, we employed Insl6 gain or loss of function mice to investigate the role of Insl6 in a T cell-mediated model of experimental autoimmune myositis (EAM). EAM models in rodents have involved immunization with native myosin-binding protein C with complete Freund’s adjuvant (CFA) emulsions and pertussis toxin (PT) [9,10]. In murine EAM, the infiltration of CD4 and CD8 T cells is observed, similar to what is seen in human PM and DM [11]. Of interest, depletion of both CD4 and CD8 T cells suppresses the myositis phenotype in this model [11]. Using this model, we find that Insl6-deficiency led to greater muscle damage, increased inflammation and reduced motor function in the murine model of EAM. Conversely, Insl6 overexpression diminished inflammation and improved motor function.

## Methods

### Materials

RPMI-1640 medium, DMEM, FBS, penicillin-streptomycin mixture, and Alexafluor 488^®^ goat anti-rabbit IgG antibody were obtained from Invitrogen (Carlsbad, CA). Anti-CD4 and anti-CD8 antibodies conjugated with eFluor^®^ 650NC were purchased from eBioscience. Fluorescein isothiocyanate (FITC)-conjugated anti-CD4 antibody was obtained from BD Biosciences (San Jose, CA). Freund’s complete adjuvant (CFA), PT, phytohemagglutinin (PHA), phorbol 12-myristate 13-acetate (PMA), protease inhibitor cocktail, RIPA buffer, and anti-laminin antibody were purchased from Sigma (St. Louis, MO). Tubulin-α antibody and horseradish peroxidase (HRP)-conjugated anti-rabbit antibodies were purchased from Cell Signaling (Danvers, MA). ^3^H-thymidine was purchased from PerkinElmer (Waltham, MA).

### Mice

MCK-Insl6-TG mice in a C57BL/6 background were generated as previously described [8]. These mice express murine Insl6 cDNA from a 4.8 kb pair fragment of the murine creatine kinase M promoter. Insl6-deficient mice were described previously [12]. In Insl6-deficient mice, exon 1 of Insl6 genomic DNA is replaced by the PGK-neomycin(Neo) selection cassette. Insl6^+/-^ heterozygous female and male pairs were used for breeding, and the offspring littermates (Insl6^+/+^ and Insl6^-/-^) were used for experiments. In some instances, C57BL/6 mice were purchased from Charles River Laboratories (Cambridge, MA). Mice were housed on a fixed 12-hour light/dark cycle and fed a normal chow diet (Teklad Global 18% protein rodent diet, 2018, Harlan Teklad). Study protocols were approved by the Institutional Animal Care and Use Committee at Boston University.

### Fc tagged Insl6 plasmid

The human IgG1 Fc was fused at the N-terminus of full-length mouse Insl6. In brief, mouse Insl6 cDNA was PCR amplified and cloned into pLEV113 mammalian expression vector (LakePharma, Belmont, CA) downstream of an in-frame human IgG1 Fc domain and a secretion signal peptide. This plasmid was used to perform hydrodynamic injection.

### Recombinant human skeletal muscle myosin-binding C protein

The recombinant skeletal muscle myosin-binding C protein was prepared using a prokaryotic expression procedure. A cDNA fragment of human fast-type skeletal muscle C protein was amplified from human skeletal muscle cDNA using PCR. Primers sequences were previously described by Sugihara *et al*. [11]. The cDNA fragment was sub-cloned into Qiagen pQE30 expression vector and the plasmid was maintained and amplified in *E. coli* XL-1 Blue strain. *E. coli* M15 (pREP4) contained in the QIAexpressionist™ was used to prepare the recombinant C protein according to the manufacturer’s protocol (Qiagen, Valencia, CA). The soluble recombinant protein was dialyzed against 0.5 M arginine, 2 mM reduced glutathione, and 0.2 mM oxidized glutathione in PBS, pH 7.4. Endotoxin removal was performed with Detoxi-Gel. The molecular weight of C protein is approximately 35 kDa.

### Chronic inflammatory myositis model

Female mice, eight to ten weeks old, were immunized intradermally with 600 μg of the C protein emulsified in CFA containing 100 μg of heat-killed *Mycobacterium butyricum* (Sigma, St. Louis, MO). The immunogens were injected subcutaneously at multiple sites around the torso or on the feet, and 2 μg of PT in PBS was injected intraperitoneally at the same time.

### Cardiotoxin-induced muscle injury model

A 10 μM solution of cardiotoxin (CTX) or equal volume of PBS was injected into the tibialis anterior (TA) muscle at 2 μl/g body weight using an insulin syringe as described previously [8]. The needle was inserted parallel to the muscle fiber longitude until reaching the tendon which inserts into the knee and then slowly withdrawn while simultaneously injecting the CTX solution in its path.

### Hydrodynamic delivery of plasmid DNA

Mice were pre-warmed by heat lamp for 20 minutes, and a plastic restrainer was used to keep mice in position during injection. Ten micrograms of Insl6-Fc or control vectors were dissolved in a large volume of saline (80 μl/g body weight) and injected quickly within 8 seconds via the tail vein [13].

### Rotarod test

Muscle function was evaluated with a Rotarod device. The test was performed on each mouse by measuring the running time until the mouse fell off the rod. Mice were initially trained to acclimate them to the task, and then tested two days thereafter. The turning speed was set at 10 revolutions/minute for knockout (KO) mice and their littermate controls, and at 20 revolutions/minute for transgenic (TG) mice and controls, to better distinguish the differences between the genetically manipulated KO and TG strains of mice and control littermates. The protocol was modified from published work [14]. Each mouse was scored according to its time on the rotating rod: score 1 corresponding to 0 to 30 seconds, score 2 for 31 to 60 seconds, score 3 for 61 to 90 seconds, and so on. A maximum score of 20 was recorded for mice running more than 10 minutes.

### Treadmill test

The treadmill test employs a moving belt (Columbus Instruments, Columbus, OH). All mice were subjected to a 30 minute run on a horizontal treadmill at 12 m/minute, twice per week before the experimental analysis [13]. To assess running performance, the treadmill was initiated with a start speed of six m/minute, and speed was increased by two m/minute every five minutes for KO mice and their littermate control mice. For TG mice and littermate controls, a starting speed was ten m/minute, and speed was increased by five m/minute every five minutes. Different belt speeds were chosen to better distinguish between the running performance of test and control sets of mice when KO and TG groups were analyzed. Electrical stimulation was applied to encourage the mice to run on the belt. The total running distance was recorded after mice elected not to run for a period of 30 seconds. Study protocols were approved by the Institutional Animal Care and Use Committee at Boston University.

### Histological analysis of murine samples

Skeletal muscle tissues were embedded in OCT compound and snap-frozen in liquid nitrogen. Serial cryostat sections (8 μm) were fixed for 10 minutes in 10% formalin, and were stained with H&E (Fisher Scientific, Pittsburgh, PA) for histological analysis. The inflammatory lesion was visualized using a × 40 objective. For immunofluorescence staining, serial cryostat sections (8 μm) were fixed in cold acetone and stained with eFluor^®^ 650NC conjugated anti-CD4 or CD8 antibody (1:100 dilution), and anti-laminin primary antibodies (1:25 dilution). Alexafluor 488^®^ goat anti-rabbit IgG secondary antibody (1:200 dilution) was used to visualize laminin expression. Fifteen randomly chosen microscopic fields from three different sections in each tissue block were examined for the presence of antigen expressing cells.

### Insl6 antibody production

Insl6 rabbit polyclonal antibody against peptides 1) VPAGVSQKKGTHT, 2) QLQKKSTNKMNTF and 3) TKEEMAVACLPFVDF was custom ordered from Thermo Fisher Inc. (Pittsburgh, PA). The antibody was purified from antiserum by antigen specific affinity purification. Antiserum was passed through a column containing resin beads conjugated to the immunogenic Insl6 peptides. Antibodies were eluted using a pH gradient, collected in a neutralizing buffer and concentrated before use.

### Western blotting

Skeletal muscle tissues were homogenized in radioimmunoprecipitation assay (RIPA) buffer which was supplemented with protease inhibitor cocktail. Equal amount of proteins were loaded on 10% NuPAGE Gels (Invitrogen, Carlsbad, CA) and run as instructed by the manufacturer. The transfer to PVDF membranes was done under wet conditions. Membranes were blocked in 5% skimmed milk or BSA solution, and then incubated with primary antibodies at 4°C overnight. Membranes were washed several times in Tris-buffered saline with Tween (TBST) while agitating, and then incubated with HRP-conjugated anti-rabbit secondary antibodies at room temperature for one hour. Chemiluminescent antigen expression was captured on film by electrochemiluminescence (ECL) using a Western Blotting Detection kit (GE Healthcare, Buckinghamshire, UK.

### Quantitative real-time PCR

cDNA was produced from total RNA using ThermoScript RT-PCR Systems (Invitrogen, Carlsbad, CA). Transcript levels were determined relative to the signal from 36B4, and normalized to the mean value of samples from control mice. Primers were purchased from Integrated DNA Technology (Coralville, IA). The following primer sequences were used; mouse Insl6 exon1 (F: 5′-GCAGCTGTGCTGTTCTTGTCTGTT-3′, R: 5′-AGGACTTTGCTCCTCCATCTCGAA-3′); Neo (F: 5′-TGTCGATCAGGATGATCTGG-3′, R: 5′-CCACCATGATATTCGGCAAG-3′); mouse CD8 (F: 5′-CTGTTTTCTGCCATGAGGGACACG-3′, R: GTTCACTTTCTGAAGGACTGGCACG-3′); mouse CD4 (F: 5′-GCTGTCACAACTCCTAGCTGTCAC-3′, F: 5′-CCTCTAATTAATACACCTTTGCCATGC-3′); mouse TNF-α (F: 5′-ACAGAAAGCATGATCCGGGA-3′, R: 5′-TCTGGGCCATAGAACTGATG-3′); mouse IFN-γ (F: 5′-CACGGCACAGTCATTGAAAG-3′, R: 5′-CATCCTTTTGCCAGTTCCTC-3′); mouse 36B4 (F: 5′-GCTCCAAGGAGATGCAGCA-3′, R: CCGGATGTGAGGCAGCAG-3′).

### Jurkat T cell proliferation assay

Jurkat cells obtained from the American Type Culture Collection (Manassas, VA) were maintained in growth medium (RPMI supplemented with 10% FBS). Cells were transiently transfected with β-galactosidase (β-gal) or murine Insl6 expression plasmids (pcDNA3, Invitrogen, Carlsbad, CA) that employed the cytomegalovirus promoter. Electroporation by Gene Pulser™ (BioRad, Hercules, CA) was conducted at 250 V and 960 μF with time constant of 24 to 30 seconds. Cell proliferation was assessed by ^3^H-thymidine incorporation. Forty-eight hours after transfection, cells were incubated with ^3^H-thymidine (2.0 μCi/10^7^ cells) for four hours. Cells were washed twice with ice cold PBS and lysed with 10% sodium hydroxide. Cell lysates were suspended in liquid scintillation buffer. The incorporation of radioactivity was determined by liquid scintillation analyzer (TRI-CARB2900TR, PerkinElmer, Waltham, MA). In related experiments, C2C12 cells were incubated with Ad-Insl6 and Ad-β-gal virus vectors at a multiplicity of infection (MOI) of 250 to 500 in culture medium for 16 hours. At this time, the virus was removed by media replacement. Cell culture media was collected 48 hours after the transfection and ^3^H-thymidine (1.0 μCi/ml medium) was added. Jurkat cells were incubated in this conditioned medium for 48 hours. The incorporation of ^3^H-thymidine was measured as described above.

### IL-2 ELISA assay

PHA and PMA activate T-lymphocytes and stimulate production of IL-2 [15,16]. Jurkat cells were transiently transfected with β-gal or murine Insl6 expression plasmids (pcDNA3, Invitrogen, Carlsbad, CA) as described above. PHA and PMA were added into culture medium 24 hours after transfection. Supernatant was collected 48 hours post-transfection and IL-2 concentration was measured by human IL-2 ELISA (Quantikine ELISA, R&D Systems, Minneapolis, MN).

### Analysis in human myositis muscle biopsy samples

Skeletal muscle samples of polymyositis and dermatomyositis patients were collected by muscle biopsy at New England Medical Center, St. Elizabeth Hospital and Tufts University. The procedures were performed after obtaining informed consent from all study subjects. The mean time in storage at -80°C for control samples was 8.8 ± 0.9 years, compared to 8.1 ± 4.0 years for the polymyositis/dermatomyositis samples. Insl6 mRNA levels were determined by real-time RT-PCR using an Applied Biosystems (Woburn, MA) Model 7300 thermal cycler. Total RNA was isolated from human fresh frozen skeletal muscles (30 μg) using RNeasy mini kit or Trizol, following the manufacturers’ instructions (Qiagen, Germantown, MD or Invitrogen, Carlsbad, CA). RNA was reverse transcribed using oligo dT and primers (50 μM each) and reverse transcriptase (RT) (Multiscribe, 250 U) in the presence of 10 mM dNTP mixture in a total volume of 100 μl. First strand cDNA and PCR reactions were generated with equal amounts of starting material using the Applied Biosystems (Woburn, MA) TaqMan Kit. Following denaturation at 95°C for 10 minutes, real-time PCR was undertaken for 40 cycles of 95°C for 15 seconds and 60°C for 60 seconds. GAPDH was used as an internal control for quantification of relative expression of specific transcripts. The sequences were as follows: human Insl6 (F: 5′-GCGGCAGGTACTTGGTGAAAGAAA-3′, R: 5′-TTTGCGGGCTTTCGAACTGGTATG-3′); GAPDH (F: 5′-GAGAGATGATGACCCTTTTGGC-3′, R: 5′-CCATCACCATCTTCCAGGAGCG-3′). Histological sections of these muscle biopsy specimens from the polymyositis and dermatomyositis patients were evaluated for the degree of atrophy by a neuropathologist who was blinded to the the level of Insl6 in the tissue. The 5, 10, 20, 25 values refer the percentage of myofibers that appeared atrophic.

### Statistical analysis

All values are presented as mean ± SEM. Student’s *t*-tests were performed to assess the statistical significance of two-way analyses. For multiple comparisons, analysis of variance (ANOVA) was employed. *P*-values of less than 0.05 were considered statistically significant.

## Results

### Insl6 ablation impairs muscle regeneration

**Figure 1:**
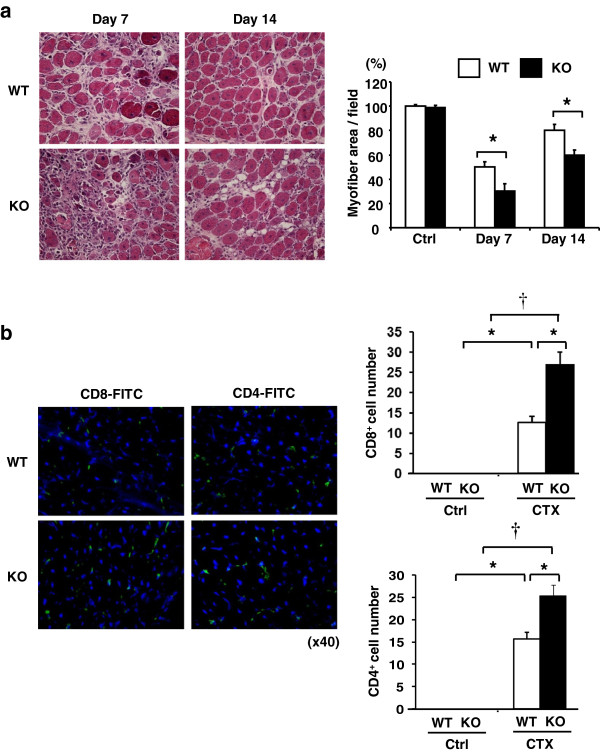
**Insl6-KO mice display impaired muscle regeneration in the model of cardiotoxin (CTX) injury. (a)** Ten micromolar solution of CTX or equal volume of PBS was injected into to tibialis anterior (TA) muscle of Insl6-KO and wild-type mice at 2 μl/g body weight with a 29-gauge needle. Representative images are H&E-stained TA muscle sections at day 7 and day 14 post-injury. Muscle regeneration was quantified in histological sections by measuring the total myofiber cross-sectional area. Data are expressed as a percentage relative to the mean value of wild-type control mice. **(b)** TA muscle sections at day 7 post-injury were stained with either fluorescein isothiocyanate (FITC)-conjugated CD4 or CD8 antibody. Nuclei were stained with 4',6-diamidino-2-phenylindole (DAPI) (blue). FITC-positive cells in 15 randomly selected fields were counted under a fluorescent microscope with 40x objective lens. The results are presented as mean ± SEM. ^*^P < 0.05, †*P* < 0.01.

Previously, we reported that overexpression of Insl6 improved the regenerative response of muscle to CTX injury [8]. To further investigate the role of Insl6 in muscle regeneration, we analyzed the response of Insl6-deficient mice (Insl6-KO) in this model. Although no muscle abnormalities were observed in the unchallenged Insl6-KO mice and muscle mass and myofiber cross-sectional area were unaffected (data not shown), following CTX delivery to the TA muscle these mice displayed an impaired regenerative response. More inflammatory cell infiltrate was observed in H&E-stained histological sections, compared to littermate wild-type mice (Figure 1a). Quantifying myofiber area in these sections revealed that the regenerative response was delayed in the Insl6-KO mice at both seven and fourteen days following CTX delivery. Since muscle injury with CTX elicits a T cell response [7], sections were also stained for CD4 and CD8 cells. CTX-treated Insl6-KO mice had increased CD4 and CD8 cells in injured muscle compared to CTX-treated wild-type littermates (Figure 1b). No CD4 or CD8 cells were observed in non-injured muscles.

### Insl6-deficiency promotes experimental autoimmune myositis severity

**Figure 2:**
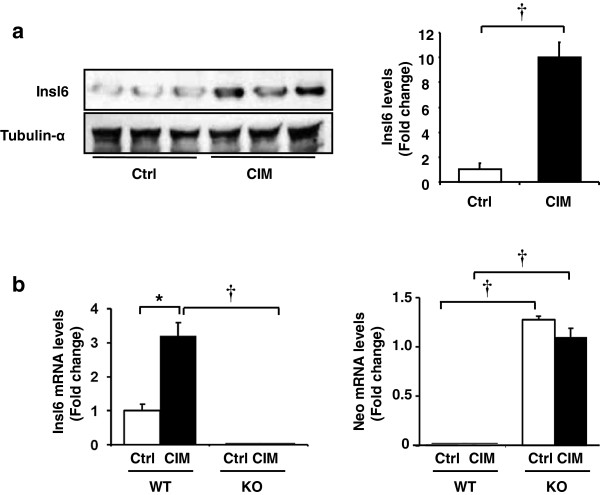
**Insl6 muscle expression is increased in a murine model of myositis.** Wild-type C57BL/6 female mice were immunized intradermally with 600 μg recombinant human skeletal muscle myosin-binding protein C or an equal volume of PBS (control) emulsified with Freund’s adjuvant. Two micrograms of pertussis toxin (PT) in PBS was injected intraperitoneally at the same time. **(a)** Gastrocnemius muscle from control and C protein-induced myositis (CIM) mice were harvested 14 days after immunization and analyzed by Western blotting for Insl6 and tubulin-α, as a loading control (left). The level of Insl6 was quantified relative to tubulin-α (right). **(b)** Transcript levels of Insl6 exon 1 and Neo cassette were measured 14 days after CIM induction in wild-type mice and Insl6-KO mice. The results are presented as mean ± SEM. ^*^P < 0.05, †*P* < 0.01.

The consequences of Insl6-deficiency were evaluated in a murine model of myositis, where a recombinant fragment of human fast-type skeletal myosin-binding C protein was used as antigen in wild-type C57BL/6 female mice [11]. In this model of C protein-induced myositis (CIM), Insl6 protein levels were upregulated at 14 days after immunization. Western blot analysis of gastrocnemius muscle (GA) revealed an approximate ten-fold upregulation of Insl6 compared with expression in muscle from untreated control mice (Figure 2a). CIM also led to an approximate three-fold increase in the expression of the Insl6 transcript (Figure 2b). No Insl6 expression was detected in muscle from the Insl6-KO mice. The Insl6-KO mice have a Neo cassette inserted, expressed from the mPGK1 promoter, to replace Insl6 exon1 [12]. This Neo cassette could be detected in Insl6-KO mice but not in wild-type mice (Figure 2b).

**Figure 3:**
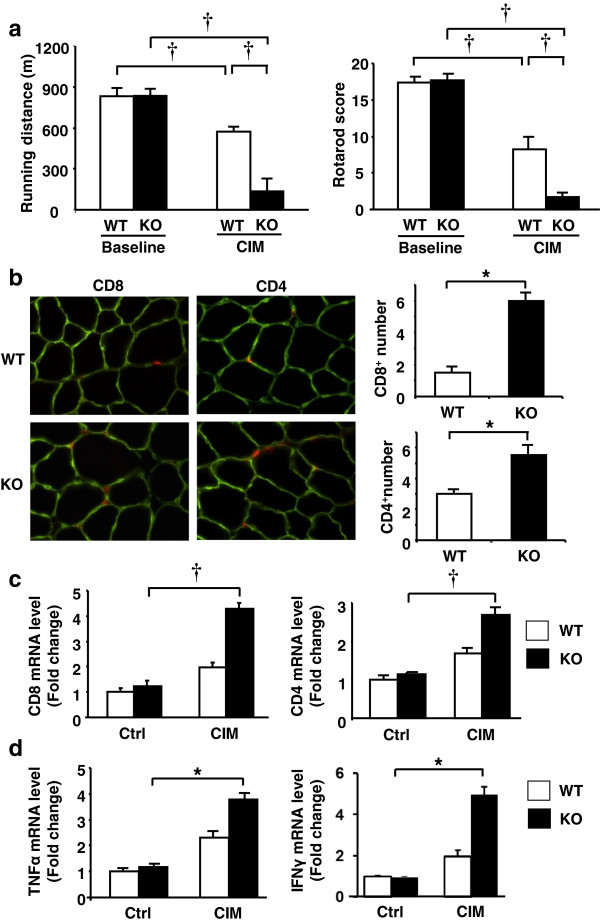
**Insl6-deficiency exacerbates experimental autoimmune myositis.** Myosin-binding C protein fragment, Freund’s adjuvant and pertussis toxin (PT) were injected into homozygous Insl6-KO female mice and their wild-type littermates. **(a)** C protein-induced myositis (CIM) and control mice were examined on treadmill and Rotarod tests before and 14 days after the immunization. For the treadmill evaluation, a starting speed of six m/minute was set and increased by two m/minute every five minutes (left). The Rotarod test was performed at ten revolutions/minute for knockout (KO) and littermate wild-type mice. Running ability and Rotarod score were measured as described in Methods. **(b)** Representative image of tibialis anterior (TA) muscle sections stained with anti-CD4 and anti-CD8 eFluor^®^ 650NC antibodies at day 14 after the immunization (left). The number of CD8 or CD4 cells per field was counted in 15 randomly selected fields. Quantitative data are presented as CD8 or CD4 cells per high-powered field (right). **(c and d)** Total RNA was isolated from lower limb muscles of control and CIM mice at post-immunized day 14, and cDNA was synthesized. Relative transcript expression of CD4, CD8, TNF-α and IFN-γ was measured by qRT-PCR. The data were normalized by 36B4 (n = 8, in each group). The results are presented as mean ± SEM. ^*^P < 0.05, †*P* < 0.01.

Muscle motor function was evaluated by the performance on Rotarod and treadmill devices in the different experimental groups. These physiological parameters did not differ between wild-type and Insl6-KO mice at baseline (Figure 3a). CIM conditions led to a running distance decline of 70 to 80% in Insl6-KO mice, whereas wild-type mice had only a 30 to 40% reduction. The CIM-induced decline in Rotarod performance was highly notable in the Insl6-KO versus wild-type mice (Additional file 1: Figure S1). Under these test conditions, Insl6-deficiency led to an approximately 90% decline in performance but wild-type mice showed an approximately 50% decline (Figure 3a).

Infiltration of CD4 and CD8 T cells in muscle is a hallmark of the CIM model [11]. Thus, CD4 and CD8 T cell infiltration was assessed in multiple lower limb muscles, including TA, GA, and quadriceps. No appreciable T cell infiltration was observed in wild-type or Insl6-KO mice at baseline (data not shown). The infiltration of CD4 and CD8 T cells was observed in most of the limb muscles of the CIM mice. In Figure 3b, a representative image of TA muscle section stained with CD4/CD8 and laminin is shown. Quantitative analyses revealed more CD4 and CD8 T cells present in the Insl6-KO muscle, compared with wild-type controls. T cell infiltration was observed in the perimysial and perivascular areas. No obvious endomysial CD8 T cell infiltration was observed in any experimental condition, in contrast with a prior report [11]. Analysis of histological sections of muscle with the macrophage marker F4/80 showed a trend toward greater infiltration of macrophages in the Insl6-KO mice under CIM conditions, but this difference was not statistically significant (Additional file 2: Figure S3).Using RNA isolated from lower limb muscles, qRT-PCR analyses were performed to measure the expression of inflammatory marker genes. Consistent with the histological analyses in Figure 3b, CD4 and CD8 transcript levels were elevated in Insl6-KO mice relative to wild-type in the CIM model (Figure 3c). Transcript levels of inflammatory cytokines TNF-α and IFN-γ were also significantly increased in Insl6-KO versus wild-type mice in the CIM condition (Figure 3d). At baseline, transcript levels of CD8, CD4, TNF-α, and IFN-γ are very low in both Insl6-KO and wild-type mice, and there was no difference between groups.

### Transgenic Insl6 overexpression ameliorates experimental autoimmune myositis

**Figure 4:**
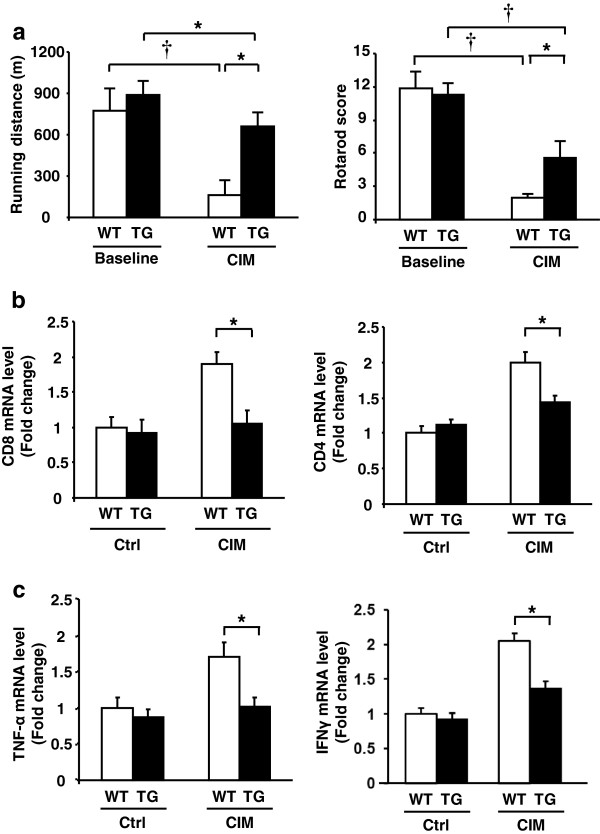
**Transgenic Insl6 over-expression ameliorates experimental autoimmune myositis.** MCK-Insl6-TG female mice and wild-type littermates were subjected to the C protein-induced myositis (CIM) protocol. **(a)** Mice were examined on treadmill (left) and Rotarod (right) before and 14 days after the immunization. For the treadmill evaluation, a starting speed of ten m/minute was set and increased by five m/minute every five minutes. The Rotarod test was performed at 20 revolutions/minute for transgenic (TG) mice and wild-type littermates. Running distance and Rotarod score were measured as described in Methods. **(b and c)** Total RNA was isolated from lower limb muscles of control and CIM mice 14 days after immunization, and cDNA was synthesized. Relative transcript expression of CD4, CD8, TNF-α, and IFN-γ (n = 5 to 7 in each group) was measured by qRT-PCR. The data were normalized by 36B4. The results are presented as mean ± SEM. ^*^P < 0.05, †*P* < 0.01.

Unchallenged Insl6-TG mice and wild-type littermates displayed similar physical performance by Rotarod and treadmill tests (Figure 4a). At 14 days after CIM induction, both Insl6-TG and wild-type mice showed decreased running and balance performance. However, the impairment occurred to a lesser extent in TG mice (Figure 4a). In the treadmill text, the running distance was reduced by 80% in wild-type mice under CIM conditions, whereas TG mice only showed a 40% reduction. In the Rotarod test, TG mice were visibly more capable of staying on the rotating cylinder longer than wild-type mice (Additional file 3: Figure S2).

Analyses of limb muscle revealed that transgenic overexpression of Insl6 diminished inflammation in the CIM model. Muscle transcript levels of CD4, CD8, TNF-α and IFN-γ increased in both wild-type and TG mice in the CIM model, but the induction of each of these transcripts was significantly less in Insl6-overexpressing mice (Figure 4b, c). However, analysis of histological sections of muscle with the marker F4/80 did not show a difference in macrophage infiltration between wild-type and TG mice (Additional file 2: Figure S3).

### Hydrodynamic Insl6 gene delivery ameliorates experimental autoimmune myositis

**Figure 5:**
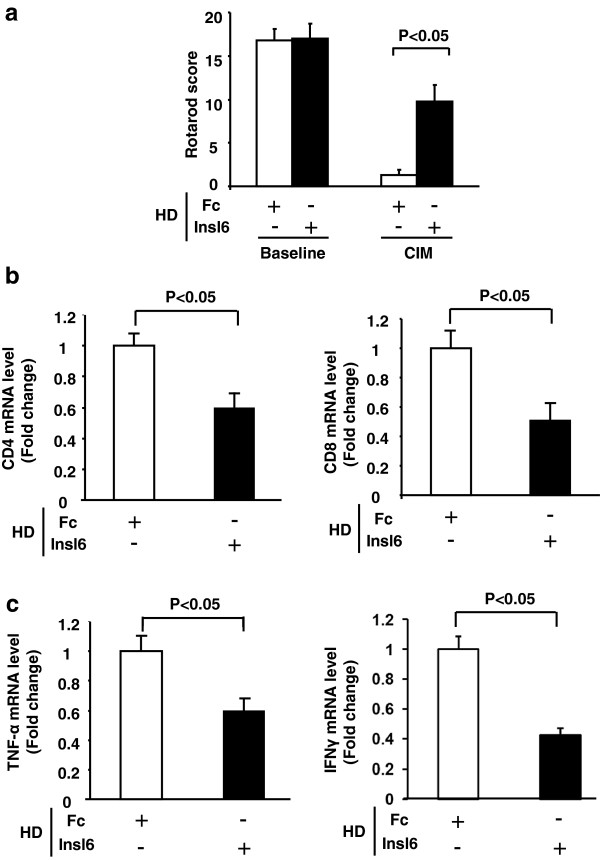
**Hydrodynamic Insl6 gene delivery improves myositis in Insl6-KO mice.** Ten micrograms of Insl6-Fc or Fc control plasmid vectors were dissolved in a large volume of saline and injected quickly via the tail vein as described in Methods. C protein-induced myositis (CIM) immunogens were injected the next day. **(a)** Mice were examined on Rotarod before and 14 days after CIM induction. The Rotarod test was performed at 10 revolutions/minute and the score was determined as described in Methods. **(b and c)** Total RNA was isolated from lower limb muscles at 14 days after CIM induction and subjected to cDNA synthesis. Relative transcript expression of CD4, CD8, TNF-α, and IFN-γ (n = 6 in each group) was measured by qRT-PCR. The data were normalized by 36B4. The results are presented as means ± SEM.

To test whether the acute expression of Insl6 is protective in the myositis model, we employed a hydrodynamic model of Insl6 gene delivery. Hydrodynamic gene delivery involves the rapid injection of a relatively large volume of plasmid DNA solution via the tail vein, leading to the transduction of liver and the production and secretion of plasmid-encoded proteins [13]. This method of acute gene delivery avoids the confounding aspects of viral vectors, in particular the activation of a T cell inflammatory response [17]. Using hydrodynamic delivery, a plasmid expressing Insl6 or an empty vector was delivered to Insl6-KO mice under non-CIM conditions at the time of immunization with the C-protein fragment. This procedure led to detectable levels of the Fc-Insl6 fusion protein in the serum, with levels as high as 1,800 ng/ml as measured by human Fc ELISA (not shown). Delivery of the Insl6-expressing plasmid had no effect on baseline Rotarod performance in wild-type mice (Figure 5a). However, plasmid-mediated Insl6 led to marked improvements in Rotarod performance in the CIM model. Hydrodynamic delivery of Insl6 plasmid to CIM mice also led to statistically significant reductions in transcript levels of inflammatory marker proteins in TA muscle, including CD4, CD8, TNF-α and IFN-γ, relative to muscle from animals that received the control vector (Figure 5b, c).

### Insl6 inhibits Jurkat T cell activation

**Figure 6:**
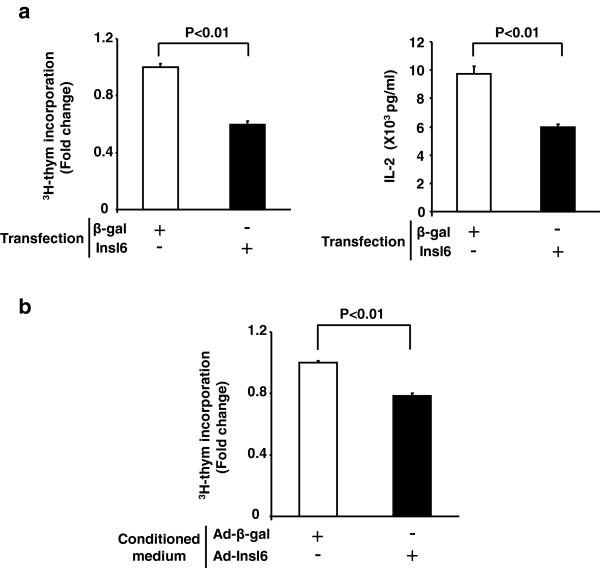
**Insl6 inhibits Jurkat cell proliferation and IL-2 secretion induced by phorbol 12-myristate 13-acetate/phytohemagglutinin (PMA/PHA) stimulation. (a)** Insl6 or β-gal encoding plasmid vectors were introduced into Jurkat T cells by electroporation. ^3^H-thymidine pulse labeling was performed 48 hours after the transfection (left). Data are expressed as relative to the mean of the β-gal experimental group (left). PMA (50 ng/ml) and PHA (1 μg/ml) were added to the culture medium 24 hours after the transfection. Cell culture medium was collected after 24 hours of PMA/PHA incubation. IL-2 concentration in the supernatant was determined by ELISA (right). **(b)** Differentiated C2C12 myotubes were transduced with Ad-β-gal or Ad-Insl6. Conditioned medium was harvested 48 hours after adenovirus transfection. Jurkat cells were incubated with this conditioned medium for 24 hours in the presence of ^3^H-thymidine. Incorporated ^3^H-thymidine was quantified. Results are presented as mean ± SEM.

Jurkat cells, an immortalized line of human T lymphocytes, were used to probe the direct actions of Insl6 on T cell signaling. Cells were transduced with Insl6 or β-gal expression vectors using an electroporation method. As shown in Figure 6a, Insl6 overexpression significantly inhibited Jurkat cell proliferation by approximately 40% as measured by ^3^H-thymidine incorporation.

IL-2 signaling promotes proliferation, survival and cytokine production in T cells [18]. Little or no IL-2 is produced from Jurkat cells at baseline, but PHA- and PMA-induced protein kinase C activation can robustly stimulate IL-2 production [19]. As shown in Figure 6a, Insl6 overexpression reduced the PMA/PHA-induced IL-2 secretion by 40%. Since Insl6 is a secreted protein [8,20], we also tested the ability of culture medium containing Insl6 to inhibit Jurkat cell proliferation. Myogenic C2C12 cells were transduced with adenoviral vectors expressing murine Insl6 [8] or a control vector expressing β-gal. Cell culture medium was collected 48 hours post-transduction. mRNA was extracted from C2C12 cell lysates and Insl6 transcript was measured by quantitative RT-PCR to confirm the Insl6 overexpression (data not shown). Jurkat cells were incubated with Insl6 conditioned medium for 24 hours, and cell proliferation was measured by the amount of incorporated ^3^H-thymidine. As shown in Figure 6b, incubation of cells with Insl6-conditioned media led to a significant reduction in proliferation.

### Reduced Insl6 expression in clinical inflammatory myositis

**Figure 7:**
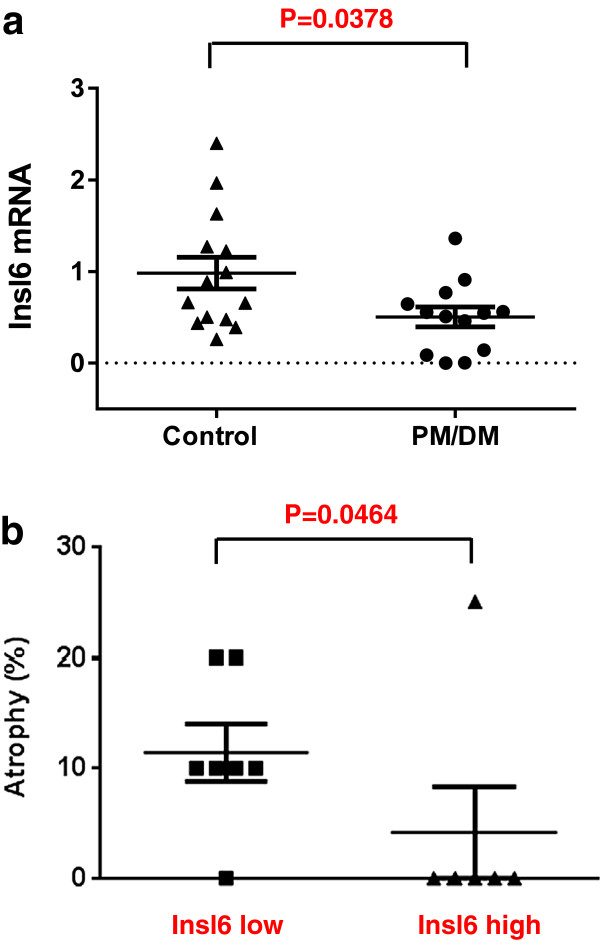
**Insl6 mRNA levels are down-regulated in human polymyositis/dermatomyositis patient skeletal muscle samples.** Human skeletal muscle biopsy samples were obtained from clinically diagnosed polymyositis/dermatomyositis (PM/DM) patients (n = 13). Non-myositis patient samples were used as a control (n = 14). The age and gender were matched in two groups. Total RNA was isolated and cDNA was synthesized. Relative transcript expression of human Insl6 was measured by qRT-PCR. The data were normalized by GAPDH. **(a)** Insl6 mRNA transcript levels are compared between control group and PM/DM patients group. Control group: 0.9825 ± 0.1725 (mean ± SEM), values (minimum 0.2621, median 0.7743 and maximum 2.402), PM/DM group: 0.5043 ± 0.1077 (mean ± SEM), values (minimum 0.0011, median 0.5471 and maximum 1.361). **(b)** Muscle atrophy was observed in tissue sections of PM/DM muscle biopsy samples following Gomori’s trichrome staining. The samples were rated based upon percentage atrophy of type 1 and type 2 fibers. PM/DM patients were divided into two groups based upon their Insl6 mRNA levels, and classified as the Insl6-low group (value ≤ median) and the Insl6-high group (value > median).

The mRNA transcript level of Insl6 was assessed in human skeletal muscle biopsy samples from polymyositis and dermatomyositis patients. The cases were identified using published criteria [21]. None of the polymyositis/dermatomyositis patients were receiving steroid therapy at the time of biopsy. Disease severity was similar in this series of inflammatory myositis cases as judged by reductions in quantified mean myofiber diameter and histopathology grading [22] compared to previous case series [23,24]. Control cases underwent biopsy for various symptoms but no individuals had high creatine phosphokinase (CPK) values and no abnormalities were found after extensive microscopic review including a special histochemistry battery. Cases of inclusion body myositis were excluded from this set. The control set had a mean age of 58.8 ± 40 (36 to 85) years at the time of biopsy, 6 male and 8 female. The polymyositis/dermatomyositis set had a mean age of 53.6 ± 5.2 (22 to 78) years, 4 male and 9 female. Differences in age, sex and time in storage were not significantly different between control and myositis groups (Table 1 and data not shown). Whereas transcript levels of Insl6 in control muscle were fairly uniform, myositis patients displayed an overall reduction in Insl6 transcript levels (*P* = 0.0378) and the expression of Insl6 was more heterogeneous with some patients exhibiting very low levels of this factor (Figure 7a). Furthermore, histological sections from the patients were scored for the degree of myofiber atrophy and then plotted against Insl6 transcript levels that were above and below the mean. A statistically significant difference in Insl6 levels relative to clinical severity was observed (Figure 7b).

**Table 1:** Characteristics of patients and control individuals

	**Control (n = 14)**	**PM/DM (n = 13)**	***P*-value**
Age, mean ± SEM	58.8 ± 4.0 (36 to 85)	53.6 ± 5.2 (22 to 78)	0.59
Male, % (n)	42.9 (6/14)	30.7 (4/13)	
Female, % (n)	57.1 (8/14)	69.2 (9/13)	
Insl6 relative transcript, mean ± SEM	0.98 ± 0.17	0.50 ± 0.11	0.038

DM = dermatomyositis; PM = polymyositis.

## Discussion

Insl6 is upregulated in injured muscle and it has been reported that it has muscle-regenerative and anti-inflammatory properties when overexpressed in the cardiotoxin model of muscle injury [8]. In this report, we demonstrate that mice lacking Insl6 display enhanced muscle inflammation and impaired regeneration following exposure to cardiotoxin. The present study also reports that Insl6 is protective in a murine model resembling human inflammatory myopathy using genetically modified mice either lacking or overexpressing Insl6. Furthermore, it is shown that overexpression of an Fc-Insl6 hybrid protein following hydrodynamic plasmid delivery improves overall muscle pathology in this model based upon functional, histological and biochemical evidence. These data support a therapeutic role for Insl6 in inflammatory myopathies where there is a high unmet need for new therapies.

Experimental myositis has been induced previously in different species with a variety of agents. Here, we employed a model of autoimmune myositis in C57BL/6 mice by administering a single immunization of recombinant human skeletal C-protein fragment emulsified in Freund’s adjuvant with heat-inactivated *M. butyricum*[11]. The resulting histopathologic abnormalities resembled some features of human polymyositis including CD4+ and CD8+ T cell infiltration. In our analyses, CD4+ and CD8+ T cells were observed in perivascular and perimysial areas, but no pronounced myofiber invasion was observed. While there was no apparent myofiber necrosis, we observed pronounced muscle motor dysfunction in both Rotarod and treadmill within two weeks of immunization. Deficiency of Insl6 led to an increase in CD4 and CD8 T cell infiltration, increased inflammatory cytokine expression and greater motor function impairment in the myositis model. Conversely, overexpression of Insl6, either by chronic overexpression in muscle or acute systemic overexpression, minimized T cell infiltration and cytokine expression in muscle and improved performance in the Rotarod and treadmill tests.

It is widely recognized that subsets of T cells play important roles in the development of the myositis phenotype [3,25-27]. In our study, Insl6 was shown to inhibit T cell activation and IL-2 cytokine secretion. These effects could be shown either by directly overexpressing Insl6 within Jurkat T cells or by treating these cells with Insl6-conditioned media. In addition, Insl6 negatively regulated the production of inflammatory cytokines in muscle. Previously, we reported that Insl6 can act on myoblasts to promote a regenerative response in muscle [8]. Thus, it appears that Insl6 can act both on inflammatory and muscle cells to control the response to injury in muscle.

Interestingly, Insl6 mRNA transcript levels are down-regulated in human myositis biopsy samples, whereas this factor is upregulated in the murine models of myositis and cardiotoxin injury. We speculate that Insl6 is an endogenous immunosuppressant or immunosurveillance factor. In animal injury models that are relatively short-term, the release of Insl6 may function as a self-protective mechanism that minimizes immune-mediated tissue destruction. However, in chronic human disease, the reduction of Insl6 by an as-yet-unidentified mechanism may contribute to the development of idiopathic inflammatory myopathies or other autoimmune diseases. Consistent with this notion, low levels of Insl6 correlated with disease severity. Thus, reestablishing the normal upregulation of Insl6 in muscle of myositis patients may have therapeutic utility.

Comparative genomic analysis shows that Insl6 is a member of the insulin-like/relaxin family of proteins that have diverse roles in reparative and reproductive processes [28]. Whereas insulin-like growth factor (IGF) and insulin activate tyrosine kinase receptors, most insulin-like family proteins signal through G-protein-coupled-receptors to control cAMP-mediated signaling events [29]. However, the receptor for Insl6 has yet to be identified, and nothing is known about the downstream signaling molecules that respond to this ligand. Related family members, including Insl1 (which is also referred to as relaxin), have been shown to have both muscle- and cardiovascular-protective actions. It has previously been shown that relaxin will promote muscle repair in a laceration model through its ability to diminish fibrosis and promote satellite cell mobilization in both young and aged mice [30,31]. Human recombinant relaxin-2 protein (RLX030) is in clinical development for the treatment of acute heart failure [32]. In the RELAX-AHF phase III clinical trial, patients treated with relaxin-2 for 48 hours experienced improved heart failure symptoms and a 37% reduction in mortality, leading to a breakthrough therapy designation by the Food and Drugs Administration (FDA). Finally, Insl3 has been shown to have cardiac regenerative properties in a zebrafish model [33]. Thus the insulin-like/relaxin family members represent a potentially interesting source of factors for the treatment of heart failure and muscle weakness, two processes that are inter-related in the pathological condition that is referred to as cardiac cachexia.

In contrast to the well-described anabolic functions of the IGF isoforms [34,35], Insl6 overexpression neither stimulates myogenesis in normal muscle nor induces C2C12 cell hypertrophy in culture [8]. Insl6 promotes muscle regeneration only after injury, and this effect is associated with an increase in satellite cell activation and the diminution of inflammatory marker expression [8]. Consistent with these observations, the Insl6-deficient mice generated by Burnicka-Turek and colleagues [12] have morphologically and functionally normal muscle at baseline. However, after muscle stress or damage, significantly impaired regeneration and deteriorated pathology were observed in the Insl6-deficient animals.

Many of the immunopathogenic processes behind myositis remain poorly understood. However, it is generally accepted that cytokines and chemokines are essential regulators of leukocyte activation and migration [3,11,36]. In animal EAM models, it has been reported that IL-6 or IL-1 blockade diminishes the severity of myositis [37]. Regulatory T cells (Tregs) have been identified as a pivotal cell population in the control of autoimmunity [38]. Treg-deficiency causes over-proliferation of lymphocytes and multi-organ autoimmunity in both humans and mice. Treg abundance is significantly reduced in skin lesions and peripheral blood of patients with dermatomyositis [39]. In Treg-depleted mice, a more severe phenotype was observed in the experimental autoimmune myositis model, whereas the injection of Tregs at the time of immunization significantly improved the disease [25].

To date, evidence for the utility of Insl6 as a potential therapy for inflammatory myositis has been limited to experimental settings using genetic models and plasmid-driven overexpression *in vivo*. Future studies will involve the development of a well-characterized, biologically-active recombinant form of Insl6 to assess the pharmacological activity of this protein and further elucidate its mechanism of action.

## Conclusions

Using mouse genetic models of Insl6-deficiency and overexpression, this study has identified a role for Insl6 in experimental autoimmune myositis. Insl6 improves skeletal muscle function and reduces inflammation in this murine model. *In vitro* studies revealed that Insl6 inhibits T cell proliferation and activation. Alterations in Insl6 expression in samples taken from both human and murine myositis-affected muscle highlight the potential for translational impact. Further investigation into the anti-inflammatory and muscle regenerative pathways mediated by Insl6 is warranted.

## Abbreviations

ANOVA: analysis of variance; β-gal: β-galactosidase; CTX: cardiotoxin; CIM: C protein-induced myositis; CFA: complete Freund’s adjuvant; CPK: creatine phosphokinase; DAPI: 4',6-diamidino-2-phenylindole; DM: dermatomyositis; DMEM: Dulbecco’s modified Eagle medium; EAM: experimental autoimmune myositis; ECL: electrochemiluminescence; ELISA: enzyme-linked immunosorbent assay; FBS: fetal bovine serum; FDA: Food and Drug Administration; FITC: fluorescein isothiocyanate; GA: gastrocnemius; H&E: hematoxylin and eosin; HRP: horseradish peroxidase; IGF: insulin-like growth factor; IFN: interferon; IL: interleukin; Insl6: Insulin-like 6; Insl6-KO: Insl6-deficient mice; KO: knockout; MOI: multiplicity of infection; PCR: polymerase chain reaction; Neo: neomycin; PMA: phorbol 12-myristate 13-acetate; PBS: phosphate-buffered saline; PHA: phytohemagglutinin; PM: polymyositis; PT: pertussis toxin; RIPA: radioimmunoprecipitation assay buffer; RLX030: human recombinant relaxin-2 protein; RT: reverse transcriptase; TA: tibialis anterior; TBST: Tris-buffered saline with Tween; TG: transgenic; TNF: tumor necrosis factor; Tregs: regulatory T cells; WT: wild-type.

## Competing interests

The authors declare that they have no competing interests.

## Authors’ contributions

LZ, SM, KN, JPD, IMA, H-KL conducted the research. XZ and HQ advised and interpreted experiments. KW oversaw the research. All authors read and approved the final manuscript.

## Supplementary Material

Additional file 1: Figure S1 Video of Rotarod evaluation of experimental autoimmune myositis in Insl6 homozygous (KO) mice versus wild-type (WT) littermates. Mice were immunized with recombinant human skeletal muscle C protein as described in the CIM methods. Balance and coordination were evaluated with the Rotarod device 14 days after CIM induction. The Rotarod test was performed by measuring time until the mouse fell off the rod. The detailed protocol and scoring method are described in the Methods. Click here for file

Additional file 2: Figure S3 Macrophage infiltration to TA muscle in experimental autoimmune myositis. Representative image of TA muscle sections stained with antibodies of anti-F4/80 (secondary antibody: Alexa Fluor594^®^) and anti-WGA-Alexa Fluor 498^®^ at day 14 after the immunization. The number of F4/80 positive cells per field was counted in five randomly selected fields in each mouse. Quantitative data are presented as F4/80 positive cells per field at x40 magnifying power (KO: n = 3, WT: n = 3, lower left), (WT: n = 4, TG: n = 3, lower right). Click here for file

Additional file 3: Figure S2 Video of Rotarod evaluation of experimental autoimmune myositis in MCK-Insl6-TG versus wild-type (WT) littermates. Details are as in Additional file 1: Figure S1 and Methods sections. Click here for file
