# Supplementary figures and images for: The injury-induced myokine insulin-like 6 is protective in experimental autoimmune myositis
Source: Skelet Muscle. 2014 Aug 4;4:16. doi: 10.1186/2044-5040-4-16 (PMC4144317; doi:10.1186/2044-5040-4-16)

# Supplement Figure 3

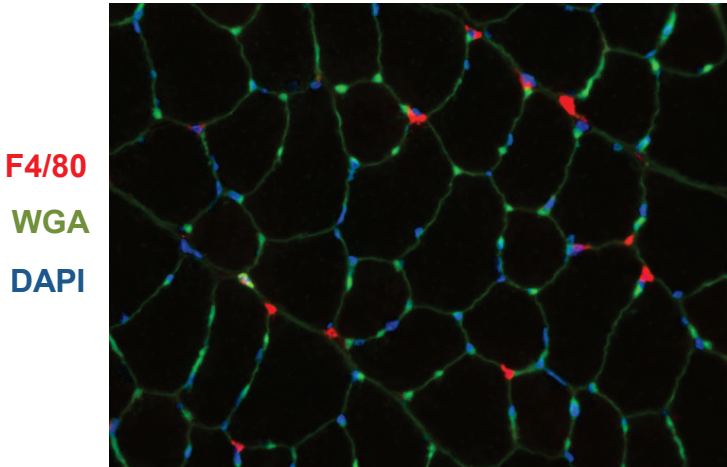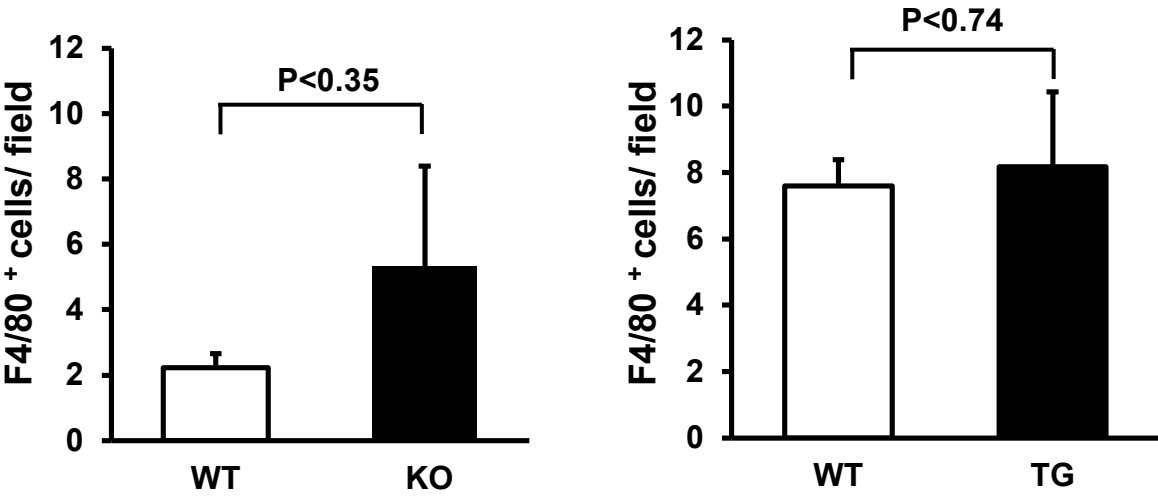

Supplement: Additional file 2: Figure S3 — Macrophage infiltration to TA muscle in experimental autoimmune myositis. Representative image of TA muscle sections stained with antibodies of anti-F4/80 (secondary antibody: Alexa Fluor594®) and anti-WGA-Alexa Fluor 498® at day 14 after the immunization. The number of F4/80 positive cells per field was counted in five randomly selected fields in each mouse. Quantitative data are presented as F4/80 positive cells per field at x40 magnifying power (KO: n = 3, WT: n = 3, lower left), (WT: n = 4, TG: n = 3, lower right). [file 2044-5040-4-16-S2.pdf]
